# Interventions for increasing chlamydia screening in primary care: a review

**DOI:** 10.1186/1471-2458-7-95

**Published:** 2007-06-04

**Authors:** Samitha Ginige, Christopher K Fairley, Jane S Hocking, Francis J Bowden, Marcus Y Chen

**Affiliations:** 1 Melbourne Sexual Health Centre, The Alfred Hospital, Melbourne, Australia; 2 Melbourne Sexual Health Centre, The Alfred Hospital, Melbourne, Australia, and School of Population Health, University of Melbourne, Melbourne, Australia; 3 School of Population Health, University of Melbourne, Melbourne, Australia; 4 Medical School, Australian National University, Canberra, Australia, and Canberra Sexual Health Centre, The Canberra Hospital, Canberra, Australia; 5 Melbourne Sexual Health Centre, The Alfred Hospital, Melbourne, Australia and School of Population Health, University of Melbourne, Melbourne, Australia

## Abstract

**Background:**

Despite guidelines recommending opportunistic chlamydia screening of younger women, screening rates in some countries remain low. Our aim was to review the evidence for specific interventions aimed at increasing chlamydia screening rates in primary care.

**Methods:**

A Medline search was conducted for controlled trials that assessed the effectiveness of interventions aimed at improving chlamydia screening rates in primary health care settings. The Medline search was done for studies in English published prior to December 2005 using the following key words: chlamydia, screening, intervention, primary care and GPs. In addition, the references cited in the articles were reviewed. Studies in English published prior to December 2005 were reviewed.

**Results:**

Four controlled studies met the inclusion criteria – 3 were randomized studies and one was not. Strategies to increase screening rates included the use of educational packages targeting primary care physicians and the correction of barriers to screening within clinic systems. In 3 studies, the intervention was associated with an increase in screening rates of between 100% and 276% (p < 0.04). In the fourth study, the intervention was associated with a significant attenuation in declining screening rates over time (4% versus 34% decline, p = 0.04).

**Conclusion:**

There are only a limited number of randomized or controlled studies that demonstrate improved chlamydia screening of younger women in primary care.

## Background

*Chlamydia trachomatis *is the most commonly reported bacterial sexually transmitted infection (STI) in the world [1]. This is of concern as untreated infection can lead to serious complications such as pelvic inflammatory disease, tubal infertility and ectopic pregnancy. Most individuals infected with chlamydia are asymptomatic [2,3], so screening is necessary to detect cases and to reduce the risk of complications. Studies suggest that selective screening for chlamydia reduces the prevalence of infection and the incidence of pelvic inflammatory disease [4,5].

Opportunistic screening of sexually active females less than 25 years of age for chlamydia in primary care has been recommended in a number of industrialized countries [6,8]. In Australia, over 80% of women aged 16–24 years visit a general practitioner (GP) at least once a year for any reason and most chlamydial infections are diagnosed in general practice [9]. However, despite the widespread availability of non-invasive testing methods for chlamydia and single dose therapy using azithromycin, chlamydia screening rates have overall remained low [9].

Ostensibly, this reflects barriers to testing that relate to both patients and health care providers. For instance, adolescents may be reluctant to seek care for their sexual health because of embarrassment or concerns about their confidentiality, while health care providers may have limited awareness of chlamydia as an issue or lack the time, knowledge and skills to manage and discuss sexual health issues [10,11]. If any chlamydia screening program is to be implemented successfully, such potential hurdles need to be identified and addressed. Preferably, this process should be evidence based. In this paper, we review studies aimed at identifying specific interventions to increase chlamydia screening rates in primary care.

## Methods

**Figure 1:**
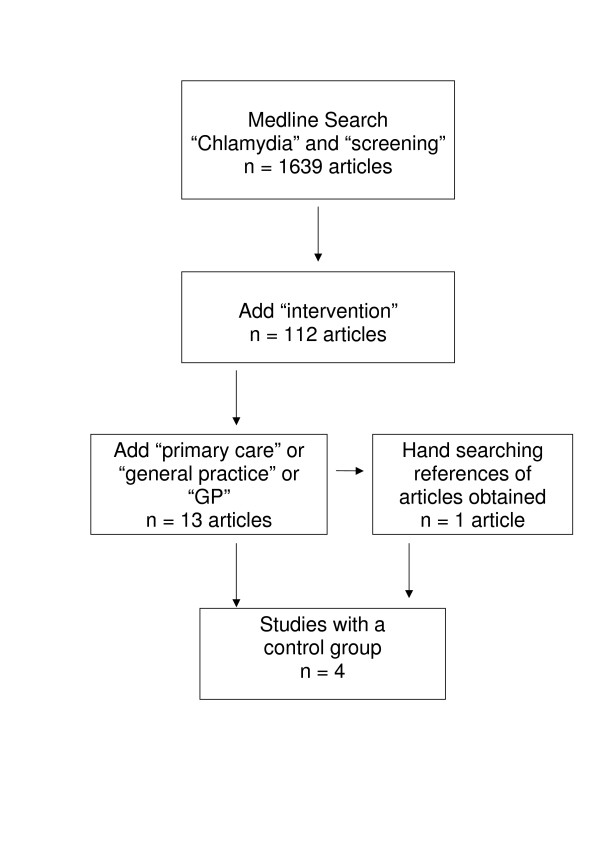
Search method used to identify controlled studies assessing interventions aimed at increasing chlamydia screening in primary care.

A Medline search was conducted in January 2006 for published, controlled trials – both randomised and non-randomised – that assessed the effectiveness of interventions aimed at improving chlamydia screening rates in primary health care settings. The Medline search was done using the following key words: chlamydia, screening, intervention, primary care and GPs. In addition, the references cited in the articles were reviewed (Figure 1).

The search was restricted to studies published in English prior to December 2005. We included studies whose specific objective was to test any intervention aimed at increasing chlamydia screening in primary care. The population of interest was patients attending primary care or general practice settings. Studies lacking a control arm were excluded. The outcome of interest was whether there was any difference in age- and sex-specific chlamydia screening rates when the intervention and control arms were compared. The selection of studies was undertaken by 2 of the authors (SG, MYC) with any discrepancies resolved through discussion and consensus among 3 of the authors (SG, MYC, CKF).

## Results

Using the search words "chlamydia" and "screening", 1,639 articles were identified and the abstracts from these articles were reviewed. Among these articles were 112 studies that recorded an "intervention". Of these, 13 were conducted in general practice or primary care. An additional study was identified through the references of one of these articles. Of the 14 studies, there were only 4 that compared screening rates using separate intervention and control groups (Figure 1). Three of these studies were randomised; one was not.

Various approaches were taken in these 4 studies to increase chlamydia screening rates (Table 1). In 3 studies, the intervention was associated with an increase in screening rates of between 100% and 276% (p < 0.04). In the fourth study, the intervention was associated with a significant attenuation in declining screening rates over time (4% versus 34% decline, p = 0.04).

**Table 1:** Controlled studies on interventions aimed at improving chlamydia screening in primary care.

Study	Setting	Intervention (duration of follow-up for testing rates)	Design	Target population	Outcomes in the intervention group	Outcomes in the control group	P value for difference
Verhoeven *et al*.^10^	36 GPs in Antwerp, Belgium	Educational package (video and text) on communication skills for sexual history taking (15 weeks)	Cluster randomized	Females patients aged <35 years	18 GPs. Median no. of females appropriately tested per GP = 6	18 GPs. Median no. of females appropriately tested per GP = 3	0.035
Shafer *et al*.^12^	10 Pediatric clinics in North Carolina	Multifaceted, system-level changes to clinical practice to overcome barriers to chlamydia screening (18 months)	Randomized	Female patients aged 14–18 years	5 clinics. 478/1017 (47%) of eligible girls screened	5 clinics. 203/1194 (17%) of eligible girls screened	<0.001
Armstrong *et al*.^13^	2 primary health centres in Scotland	Introduction of a health advisor to increase awareness and to provide training on chlamydia screening guidelines (6 months)	Non-randomized	Males and female patients aged 15–24 years	1 health centre. No. of chlamydia tests*: Pre-intervention: 152 Post-intervention: 335	1 health centre. No. of chlamydia tests*: Pre-intervention: 336 Post-intervention: 374	0.001
Allison *et al*.^14^	191 primary care physician offices in the US	Internet based continuous medical education on chlamydia screening (2 years)	Randomized	Female patients aged 16–26 years	95 offices. Screening rates: Pre-intervention: 16.2% During intervention: 13.3% Post-intervention: 15.5%	96 offices. Screening rates: Pre-intervention: 18.9% During intervention: 13.0% Post-intervention: 12.4%	0.044^†^

*Denominator values are not available. The numbers of tests quoted are for all ages.

^†^p value is for post intervention differences after adjusting for baseline performance.

In a study by Verhoeven *et al*., a cluster randomised controlled trial was carried out among Belgian GPs to assess the impact of an short educational package on chlamydia screening rates among female patients less than 35 years of age [10]. The interventional package included a short video on communication skills where a GP offers testing for chlamydia during a simulated consultation. This was accompanied by text on communication skills for taking a sexual history, which included techniques for increasing patient comfort levels. Eighteen GPs in the intervention group and 18 in the control group were entered into the analysis.

Over the 15-week study period, GPs in the intervention group performed significantly more appropriate screening tests within recommended guidelines (median of 6 patients per GP) than GPs in the control group (median of 3 patients per GP, p = 0.035; Table 1).

Shafer *et al*. undertook a cluster randomized controlled trial involving paediatric clinics in Northern California. Ten paediatric clinics were randomly assigned to provide either usual care (n = 5) or the intervention (n = 5) [12]. Staff at both the intervention and control clinics received information on chlamydia screening guidelines. In addition, the intervention clinics introduced a clinical improvement initiative aimed at overcoming barriers to chlamydia screening at all levels of clinical practice. This intervention was based on a model to change practice which consisted of 4 stages: (1) "engage", (2) "team building", (3) "redesign clinical practice", and (4) "sustain the gain".

The first of these stages involved engagement with the health maintenance organisation's leaders by presenting evidence showing the gap between current and best practice with respect to chlamydia screening. In addition, awareness among clinic staff was raised through a brief introduction to the intervention and to team building concepts. The second step consisted of the formation of adolescent care teams comprised of clinic staff who would act as champions for the project. These teams completed a workshop that emphasized skill building and implementation of a model for practice change. A toolkit was developed to facilitate incremental changes. This included a customized clinic flow chart which helped team members to identify barriers to and solutions for changing their practice. It also contained promotional material designed to raise awareness about screening adolescent girls: logos on stickers used to cue charts, on buttons worn by staff, and on pens and posters placed in the intervention clinics.

The third stage of the intervention consisted of monthly meetings of clinic team members, where chlamydia screening rates and documentation on encounters with adolescents were reviewed to assess the effectiveness of prior incremental measures aimed at boosting screening rates. Barriers to screening were identified as well as strategies to overcome these. As part of this process, all intervention clinics decided to institute universal urine specimen collection from all adolescents at clinic registration, prior to their examination. As part of the final intervention stage, the teams developed performance indicators (number of visits and chlamydia screening rates) and customized information infrastructure to assist in monitoring progress against these.

Over the 18-month study period, significantly more females aged 14–18 were screened in the intervention clinics (478 of 1017) compared with the control clinics (203 of 1194, P < 0.001; Table 1).

In a study by Armstrong *et al*., the effect on chlamydia testing within a Scottish primary health care centre was examined following the introduction of a health adviser whose role it was to raise awareness of chlamydia and to train staff on chlamydia testing guidelines [13]. The number of chlamydia tests performed during the 6 month period the adviser was present (n = 335) was higher than that for the same 6 month period during the preceding year (n = 152). For the same 6-month periods, the number of tests performed in a control clinic, where no health adviser was introduced was 336 and 374 respectively (Table 1).

The change in testing rates in the intervention centre (120%) was significantly higher than the change in testing in the control centre (11%, p = <0.001). However, denominator values – the number of patients actually seen during those periods – were not provided. Much of the increase in chlamydia testing seen in the intervention clinic occurred in patients outside the target age range (15–24 years), with no increase in actual detection rates. This was attributed to a deficiency in staff training and linkage of chlamydia screening with cervical screening, which led to a tendency towards the testing of older women.

In a study by *Allison *et al., 191 primary care physicians offices in the US were randomized either to an internet-based continuing medical education (CME) program for increasing chlamydia screening (n = 95) or to a control arm (n = 96) [14]. The intervention consisted of 4 CME modules that were released every 3 months. The modules emphasized a number of points: that young, sexually active women are at high risk for asymptomatic infection that may lead to future serious health consequences; that recently developed urine-based screening allows diagnosis without a pelvic examination; and that infection may be treated easily and effectively.

The mean chlamydia screening rates in women aged 16–26 years before, during and after the intervention for the control offices were 18.9%, 13.0% and 12.4%, respectively. For the intervention offices, they were 16.2%, 13.3%, and 15.5%, respectively (p = 0.044 for post intervention differences after adjusting for baseline performance; Table 1). The difference in post intervention screening rates by study group remained significant when adjusting for both pre intervention and intra intervention screening rates using repeated-measures analysis (p = 0.009). Thus, the intervention appeared to forestall the significant decline in screening rates seen in the control clinics.

As the authors themselves point out, the effect seen was remarkable given the limited intensity of the intervention: each physician completed on average 2.4 CME modules, each taking an average of 12 minutes to complete. Moreover, the magnitude of the real effect may have been underestimated given the study design.

## Discussion

In this review, we identified 4 published, controlled studies which assessed the efficacy of interventions aimed at increasing chlamydia screening in primary care. In all of these studies, each of which took a different approach to increasing screening, but all of which primarily targeted younger females, significant increases in chlamydia screening rates were associated with the interventions. Although there are many studies that look at possible ways of increasing chlamydia screening within health services, few of these include a control arm, which importantly allow comparison of screening rates in groups exposed and not exposed to the intervention.

The strengths and limitations of the studies included in this review varied substantially. For instance, sample sizes ranged from the 2 health care centres in the study by Armstrong *et al*. to the 191 physician offices in the study by Allison *et al*. In some of these studies, especially that by Shafer *et al*., the intervention consisted of multiple measures, each of which could have potentially influenced screening rates in their own right, and it is unclear which of these were in fact the most effective and which in practice could be dispensed with. Finally, the populations and health care systems in the study settings varied, so the extent to which the findings would apply to other settings is uncertain. Because of the diversity of the interventions in these studies, meta-analytical pooling of the data was not possible.

Despite these weaknesses, a range of potentially effective strategies were identified in these studies. These included those aimed at: increasing awareness of chlamydia and its sequelae; improving knowledge of screening guidelines and non-invasive testing; improving physicians' communication skills, including sexual history taking; and overcoming barriers within clinic systems. A number of methods were used to disseminate information and training, including written guidelines, video educational packages, and internet based CME modules.

The question remains as to which of these strategies should be employed in primary care to increase chlamydia screening. It is not clear which would be the most cost-effective and feasible, particularly in view of the competing priorities and time constraints that clinicians invariably face. While the effect of the intervention seen in the study by Shafer *et al*. appeared to be relatively large, the intensity of measures employed would most likely be difficult to implement universally. By contrast, the internet based CME modules used by Allison *et al*. would be easier to disseminate, relatively cheap and easily accessible. For clinicians without internet access, the short educational package of the type employed by Verhoeven *et al*. would be an alternative.

It is notable that the results seen in the study by *Allison et al*. differed from that in the other studies in that screening rates actually fell, albeit to a lesser extent in the intervention arm. The reasons for the overall decline in screening rates in the study are uncertain; however, they highlight the fact that screening rates may be influenced or indeed driven by external factors. For instance, overall screening rates for chlamydia may be influenced by how aware clinicians and members of the public are for the need for STI screening [15]. They are also likely to be influenced by the characteristics of the health systems in which screening is undertaken, not least by the access to screening available to people at risk for infection and by factors that affect the level of access [9]. Any programme aimed at enhancing chlamydia screening in primary care will need to take such factors into account.

Aside from the overarching objective of increasing screening rates, any chlamydia screening programme also needs to define the target population and how the programme will be implemented. Guidelines from a number of countries advocate opportunistic screening of sexually active young females under the age of 25 when they attend health services. Such an approach to screening has the advantage in that it utilizes existing infrastructure and is therefore less resource intensive than more systematic approaches such as the registry-based cervical screening programmes operating in some countries. A systematic approach to screening is likely to result in higher coverage with greater health benefits, but at a higher cost, while the success of opportunistic screening is dependent on adequate levels of awareness and training on the part of health care providers.

The optimal approach to chlamydia screening, however, remains uncertain [16]. Whether or not the inclusion of men into chlamydia screening programmes results in additional health benefits is also unknown although modeling suggests that this could be the case [17]. Any eventual programme should also consider additional strategies that could enhance chlamydia control such as measures to facilitate partner notification, testing and treatment [18].

## Conclusion

The findings in this review indicate that there are differing approaches that can be taken to support primary health care providers to significantly increase targeted chlamydia screening of younger women. However, there are really too few controlled studies to allow any comprehensive discussion of evidence based options. Given the efforts that have gone into rolling out chlamydia screening in various countries and the continuing, low screening rates that have been observed in some, further randomized, controlled studies using novel strategies are needed.

## Competing interests

The author(s) declare that they have no competing interests.

## Authors' contributions

All authors have contributed to the preparation and checking of this manuscript, and have read and approved the final version.

## Pre-publication history

The pre-publication history for this paper can be accessed here:


